# Proteomic analysis of adipose tissue during the last weeks of gestation in pure and crossbred Large White or Meishan fetuses gestated by sows of either breed

**DOI:** 10.1186/s40104-018-0244-2

**Published:** 2018-04-03

**Authors:** F. Gondret, B. Guével, M. C. Père, H. Quesnel, Y. Billon, E. Com, L. Canario, I. Louveau, L. Liaubet

**Affiliations:** 1 PEGASE, Agrocampus Ouest, INRA, 35590, Saint-Gilles, France; 2 Protim, Inserm U1085, Irset, Université Rennes 1, Campus de Beaulieu, 35042 Rennes Cedex, France; 3 GenESI, INRA, Le Magneraud, 17700, Saint-Pierre-d’Amilly, France; 4 GenPhyse, INRA, INPT, INPT-ENV, Université de Toulouse, 31320 Castanet-Tolosan, France

**Keywords:** Adipose tissue, Fetus, Genetics, Maturity grade, Proteome

## Abstract

**Background:**

The degree of adipose tissue development at birth may influence neonatal survival and subsequent health outcomes. Despite their lower birth weights, piglets from Meishan sows (a fat breed with excellent maternal ability) have a higher survival rate than piglets from Large White sows (a lean breed). To identify the main pathways involved in subcutaneous adipose tissue maturation during the last month of gestation, we compared the proteome and the expression levels of some genes at d 90 and d 110 of gestation in purebred and crossbred Large White or Meishan fetuses gestated by sows of either breed.

**Results:**

A total of 52 proteins in fetal subcutaneous adipose tissue were identified as differentially expressed over the course of gestation. Many proteins involved in energy metabolism were more abundant, whereas some proteins participating in cytoskeleton organization were reduced in abundance on d 110 compared with d 90. Irrespective of age, 24 proteins differed in abundance between fetal genotypes, and an interaction effect between fetal age and genotype was observed for 13 proteins. The abundance levels of proteins known to be responsive to nutrient levels such as aldolase and fatty acid binding proteins, as well as the expression levels of *FASN,* a key lipogenic enzyme, and *MLXIPL*, a pivotal transcriptional mediator of glucose-related stimulation of lipogenic genes, were elevated in the adipose tissue of pure and crossbred fetuses from Meishan sows. These data suggested that the adipose tissue of these fetuses had superior metabolic functionality, whatever their paternal genes. Conversely, proteins participating in redox homeostasis and apoptotic cell clearance had a lower abundance in Meishan than in Large White fetuses. Time-course differences in adipose tissue protein abundance were revealed between fetal genotypes for a few secreted proteins participating in responses to organic substances, such as alpha-2-HS-glycoprotein, transferrin and albumin.

**Conclusions:**

These results underline the importance of not only fetal age but also maternal intrauterine environment in the regulation of several proteins in subcutaneous adipose tissue. These proteins may be used to estimate the maturity grade of piglet neonates.

**Electronic supplementary material:**

The online version of this article (10.1186/s40104-018-0244-2) contains supplementary material, which is available to authorized users.

## Background

Development and growth are ongoing processes that begin at conception and continue throughout life. Physiological maturity at birth is recognized as a major determinant of neonatal survival, postnatal growth and subsequent health outcomes [1–3]. For instance, preterm newborns with a low maturity grade have difficulty adapting to extra-uterine life [4]. In pigs, among which mortality affects a significant proportion of neonates [5], lower maturity at birth is also associated with lower survival [6, 7]. Physiological maturity at birth is defined as the state of full development allowing neonatal survival; thus, it results from the sufficient development of organs, tissues and body systems during gestation. Inter-individual differences in physiological maturity may be detected by measuring body size, organ weight, energy reserves and/or cellular characteristics and functionality. Various findings support the view that the degree of adipose tissue development at birth is important for, or at least indicative of, the physiological maturity of neonates. In particular, piglets with low birth weight had lower body fat content than their appropriately grown littermates [8]. Reduced body fat content in piglets was also observed when birth weight was reduced in response to high- or low-protein gestation diets [9]. Conversely, neonates from fat-breeds sows had better survival than neonates from modern-breeds sows that have been intensively selected for lean growth rate [10, 11]. Piglets from dams selected for higher subcutaneous fat thickness [12, 13] also had higher postnatal survival. Finally, piglets with higher breeding values for birth-to-weaning survival exhibited a greater percentage of body fat at full term [6]. Taken together, these data indicated that fetal development of adipose tissue should be considered in predicting piglet survival. Fetal development of adipose tissue could also be important in predetermining postnatal growth of adipose tissue and predisposing animals to metabolic disorders in later life [14].

This study was undertaken to achieve a better understanding of the biological pathways involved in adipose tissue maturation by exploring adipose tissue features in four genotypes of pig fetuses during the last three weeks of intrauterine life. Two gestational time points (d 90 and d 110) were chosen to collect subcutaneous adipose tissue in pure and reciprocal crossbred fetuses gestated by either fat (Meishan) or lean (Large White) maternal breeds. The Meishan is a Chinese breed that produces small piglets with extremely low mortality at birth, whereas the Large White is a modern European breed exhibiting high piglet mortality [15]. Proteome profiling has been used as an effective approach to assess different biological processes in adipose tissue [16–18] and as a valuable tool for identifying traits related to metabolic adaptation and adipose dysfunction [19].

## Methods

### Animals and sample collection

A total of 48 piglets of four fetal genotypes and two gestational ages were considered, which led to eight experimental groups (*n* = 6 fetuses per experimental group) as previously described [20]. In brief, 12 Meishan (MeiS) and 12 Large White (LW) sows were inseminated with mixed semen from MeiS and LW boars, so that each litter was composed of pure (MeiS or LW) and crossbred (F1_MeiS from MeiS sows and F1_LW from LW sows) fetuses. Semen was collected from 3 LW boars and 3 MeiS boars and combined into 3 different mixtures, each derived from one boar per breed. Both parental genes and uterine environment differed between the fetuses. All sows were anesthetized at d 90 or d 110 after conception (average gestation term: 114 d). Their fetuses were quickly obtained by caesarean section. Blood (approximately 5 mL) was immediately collected from the umbilical artery via a 21-gauge needle and a 5-mL syringe and placed in heparinized tubes. Plasma was prepared by low-speed centrifugation (2,000×*g* for 10 min at 4 °C) and stored at − 20 °C until further analysis. Fetuses were euthanized by an intra-cardiac injection of 5 mg of KCl. The number, weights and sexes of the fetuses were recorded. In each litter, two male fetuses, one purebred and one crossbred, were then selected. They were chosen so that their weight was representative of the mean weight of the fetuses of the same genotype in the litter. For each of the selected fetuses, dorsal subcutaneous adipose tissue was then rapidly collected from the third lumbar vertebra to the last rib level by an incision made on the dorsal side of the body. Any residual skin fragments were carefully trimmed off the adipose tissue samples, and the adipose tissue was cut into small pieces, snap frozen in liquid nitrogen, placed into 2-mL sterile tubes and then stored at − 75 °C until analyses.

### Lipid content in adipose tissue

Triglycerides content in subcutaneous fetal adipose tissue was determined using the method described by Xu et al. [21]. Briefly, 100 mg of subcutaneous adipose tissue sample was homogenized in 5 mL of ethanol at room temperature, followed by centrifugation at 15,000 ×*g* for 10 min. Triglycerides content was determined in supernatant using an enzymatic kit (Triglycérides Enzymatique PAP 150 #61236; Biomérieux, Marcy l’Etoile, France) and a clinical chemistry analyzer Konelab 20i (Thermo Fischer Scientific, Courtaboeuf, France).

### Soluble proteins extracted from adipose tissue

Adipose tissue samples (~ 150 mg each) were homogenized in 700 μL of ice-cold buffer (pH = 8.5) containing 30 mmol/L Tris, 1 mmol/L EDTA and 0.25 mol/L sucrose (TES buffer) and protease inhibitors (Roche Complete Mini EDTA-free Protease Inhibitor Tablet, Sigma Aldrich, France). The mixture was stirred for 1 h on ice using glass bead agitators (Heidolph, Schwabach, Germany) and then centrifuged at 10,000×*g* for 15 min at 4 °C. The resulting supernatant contained soluble proteins, this protein sub-fraction allowing the majority of enzymes and some of the less-expressed proteins to be more easily studied by 2D gel electrophoresis [22]. Before proteomics analyses, extracts were concentrated using Amicon Ultra-4 10 K centrifugal filter device (Millipore, Molsheim, France) to ensure a minimal protein concentration above 2 mg/mL [17, 18]. The total protein concentration of the extracts was assessed by Bradford reagent (BioRad, Hercules, CA, USA) using bovine serum albumin as a standard. Protein extracts were stored at −75 °C until use.

### High-resolution two-dimensional gel-based differential analysis of adipose proteins

The two dimensional-differential gel electrophoresis (2D–DIGE) procedure was performed as previously described [18]. Briefly, soluble adipose proteins (50 μg) were minimally labelled with 400 pmol of amine-reactive Cy3 or Cy5 fluorescent cyanine dyes (GE Healthcare, Saclay, France) and an internal standard comprising equal amounts of each protein extract was labelled with Cy2 dye. For each gel, two samples were run with the pooled standard (Additional file 1) in 24 cm precast immobilized pH 4–7 gradient strips and using an IPGphor system (GE Healthcare). Isoelectric conditions were optimized to obtain well-resolved protein spots in 2D–gels at both ages: stepwise voltage increase with 120 V for 1 h, 200 V for 1 h, 500 V for 1 h, 1,000 V for 6 h, linear gradient up to 8,000 V within 1.5 h, and 8,000 V for 7.5 h for a total focusing of 74,300 V-h. Proteins were then separated on 12.5% SDS polyacrylamide gels using a vertical Ettan Dalt Six device (GE Healthcare). Two preparative gels (one for d 90 and one for d 110) made with 600 μg of proteins from the corresponding pooled extracts were run in the same conditions and stained with LavaPurple (Serva Electrophoresis, CliniSciences, Nanterre, France). Representative 2D–gels of fetal adipose tissue obtained at d 90 or d 110 of gestation were shown in Additional file 2.

### Gel imaging

After SDS-PAGE, the gels were scanned with a Typhoon 9400 Imager (GE Healthcare) at 200 μm resolution (pixel size) using appropriated excitation and emission wavelengths, filter and photomultiplier (PMT) sensitivity for each dye (PMT values: 415, 400 and 430 for Cy2, Cy3 and Cy5, respectively). Gel images were processed with the DeCyder software (v5.00.08; GE Healthcare), which allows quantification, gel matching and statistical analyses. First, the Differential In-gel Analysis (DIA) module co-detected the 3 images from a single gel (i.e., the internal standard as the primary image and two samples as secondary and tertiary images, respectively), measured spot volume in each image, and expressed these values as a spot ratio of the internal standard. Second, the Biological Variation Analysis (BVA) module used those images to match protein spots across comparable gels, using the internal standard for gel-to-gel matching.

### Protein identification

Preparative gels were matched with the DIGE images to edit the picking list. Spots with a very weak intensity or bad-defined in preparative gels were excluded from the list. Tentative identification was thus performed on 182 spots. These spots were excised, digested, and peptides were processed as previously described [18]. Mass fingerprints were acquired using a matrix-assisted laser desorption ionization-time (MALDI) combined with a time-of-flight (TOF)/TOF mass spectrometer (Ultraflex, Bruker Daltonics). When applicable, precursor ions in the MALDI-MS mass spectrum were subjected to fragment ion analysis in the tandem (MS/MS) mode. Lists of monoisotopic peaks were submitted to SwissProt sequence database restricted to Mammalia taxonomy (65,132 sequences) and NCBI non-redundant sequences databases of mammalian proteins (845,127 sequences) using the MASCOT search engine (version 2.2.07; http://www.matrixscience.com) and stored in ProteinScape bioinformatics platform (v1.3 SR2; Burker Daltonics). Search conditions were as follows: initial rather open mass window of 70 ppm for an internal calibration, one missed cleavage allowed, fixed modifications of cysteines by iodoacetamide, and methionine oxidation as variable modifications. Results were scored using the probability-based Mowse score. A score greater than 61 for the SwissProt database and 72 for the NCBInr database, respectively, was retained for identification. For the MS/MS experiment, the mass tolerance was set at 100 ppm for the parent ion and at 0.5 Da for the fragment ions. A score greater than 27 indicated peptides with significant homology, and scores greater than 32 (for the SwissProt database) or 43 (for the NCBInr database) indicated a significant identification. The rate of success in protein identification averaged 72% in this experiment.

### Gene-ontology based pathway analysis

Lists of identified proteins were clustered using annotation categories included in Gene Ontology (GO) Biological Process, KEGG Pathways, and PIR SuperFamily Names, which were collected in the DAVID knowledgebase (Database for Annotation, Visualization and Integrated Discovery bioinformatics tool; v6.7, https://david.ncifcrf.gov/). Orthologous gene identifiers for *Homo sapiens* were associated to the lists of identified proteins and further explored with the functional annotation clustering tool. Significance levels of functional annotations over-represented were appreciated by the modification of Fisher’s exact test (*P* < 0.05) and the enrichment calculated for individual specific terms in each functional group. To order the relative importance of the gene groups, the E score was calculated by minus log transformation of the geometric mean of the modified Fisher Exact EASE Scores of all the annotation terms that belong to the cluster.

### Expression levels of selected genes by qPCR in adipose tissue

Expression levels of encoded proteins that were identified with differential abundance due to age and(or)genotype by proteomics were investigated by qPCR. Other selected genes encode proteins that cannot be visualized on 2D gels: the fatty acid synthase (*FASN*), a whole enzymatic system composed of two identical 272 kDa multifunctional polypeptides acting in lipogenesis, and well-known transcriptional regulators of adipogenesis and lipid metabolism. Gene primers (Additional file 3) were designed using PrimerExpress software (version 3.0, Applied Biosystems, Foster City, CA, USA) from pig sequences, taking into account intron-exon organization. Samples of subcutaneous adipose tissue were homogenized in Trizol reagent (Invitrogen, Cergy-Pontoise, France) using a TissueLyser (Qiagen, Courtaboeuf, Paris) and treated as described previously [23]. Reverse transcription was performed (High capacity cDNA reverse transcription kit, Applied Biosystems, Foster City, CA) from total RNA. Gene expression was measured in triplicates on a StepOnePlus real-time PCR system (Applied Biosystems) using the Fast SYBR® Green Master Mix reagent in standard thermal cycling conditions and specific primers. For each gene, the normalized expression level N was calculated according to the following formula: N = E^-ΔCq (sample-calibrator)^/NF where the calibrator is a pool of samples, E the PCR efficiency and NF, a normalization factor. This NF factor was calculated as the geometric mean of two housekeeping genes (*HPRT1* and *PPIA*) declared as the most stables among 5 tested genes [24] by using the geNorm algorithm (https://genorm.cmgg.be/).

### Plasma concentrations of nutrients and IGF-I

Glucose, fructose, lactate were chosen as indicators of carbohydrate metabolism, and albumin was used to indicate protein metabolism. Blood parameters were analyzed using commercial kits (the Glucose RTU, Lactate PAP and Albumin kits from bioMérieux, Marcy l’Etoile, France, and the Fructose kit from Thermo Electron, Cergy-Pontoise, France) on a Konelab analyzer. In addition, the plasma concentration of insulin-like growth factor 1 (IGF-I) was determined after an acid-ethanol extraction using the IRMA IGF-I kit (Immunotech, Marseille, France). The intra-assay coefficient of variance was 7.4%, and the lower limit of detection was 7.5 ng/mL.

### Statistical analysis

Protein spots abundance, triglycerides content, expression levels of target genes in adipose tissue, and plasma concentrations of nutrients and IGF-I were compared by ANOVA for the effects of developmental age, fetal genotype and the interaction between age and genotype (A × G), by using the GLM procedure of the SAS software (SAS Institute, Cary NC, New-York). Protein spot abundance values were analyzed after logarithmic transformation to fit normal distribution. A *P*-value of less than 0.05 was used as the cut-off. Lists of spots identified with a differential abundance due to age, fetal genotype and(or) an interaction effect between age and genotype were edited in picking lists. Benjamini-Hochberg (BH) multiplicity correction of the *P*-values for a control of the FDR was also calculated. Correlation was calculated between the mean abundance of different spots identified as albumin in adipose tissue and the albumin concentration measured in plasma, by using the CORR procedure of SAS. Both correlation coefficient (*r*) and *P*-value were shown.

## Results

### Fetal weight and lipid concentration in fetal adipose tissue

**Fig. 1:**
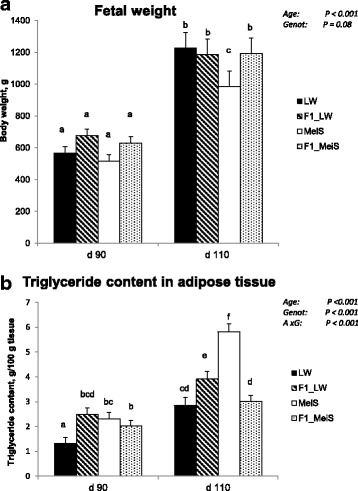
Body weight and lipid concentration in subcutaneous adipose tissue of pure and crossbred Large White or Meishan fetuses gestated by sows of either breed. Purebred Large White (LW) or Meishan (MeiS) sows were inseminated with mixed semen from LW and MeiS boars. Pairs of purebred and crossbred (F1) fetuses were excised at d 90 or d 110 of gestation (*n* = 6 per age and per genotype). LW and F1_LW were from LW sows, whereas MeiS and F1_MeiS were from MeiS sows. A total of 48 fetuses were weighed (**a**). The triglyceride concentration was measured in the dorsal subcutaneous adipose tissue (**b**). Analysis of variance was used to determine the effects of gestational age, fetal genotype and the interaction between age and genotype (A × G). Least squares means sharing a common superscript letter did not significantly differ (*P* > 0.05)

The body weight of the fetuses increased almost twofold (597 g to 1,114 g on average; *P* = 0.001) during the last three weeks of gestation (*n* = 48; Fig. 1a). Fetal weight also tended to differ (*P* = 0.08) between genotypes, owing to the lighter weight (*P* < 0.05) of MeiS fetuses at d 110. The triglycerides content of the subcutaneous adipose tissue increased between d 90 (2.03% on average) and d 110 (3.73% on average). This content was greater in pure MeiS fetuses than in pure LW fetuses at both ages (Fig. 1b), and the difference was more pronounced at d 110 than at d 90 (A × G interaction: *P* < 0.001). The triglycerides content of the adipose tissue was similar in F1_LW and F1_MeiS fetuses at d 90, whereas it was higher in crossbred F1 fetuses having MeiS paternal genes (F1_LW) than in F1 fetuses having LW paternal genes at d 110.

### A powerful experimental design to test for changes in adipose tissue proteins during gestation

**Fig. 2:**
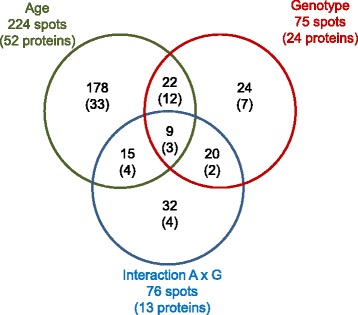
Venn diagrams for regulated protein spots in the subcutaneous adipose tissue of purebred and crossbred Large White or Meishan fetuses gestated by sows of either breed. Subcutaneous adipose tissues sampled from 4 fetal genotypes on d 90 or d 110 of gestation, were analyzed by two-dimensional differential in-gel analysis (2D–DIGE). Analysis of variance was used to determine the effects of gestational age, fetus genotype and the interaction between age and genotype (A × G). Venn diagrams were used to identify protein spots (and unique proteins) that were regulated by at least one of these factors

A total of 1,944 to 2,233 spots were detected on the 2D gels, and 75% were successfully matched across the different gels. A total of 224 protein spots had differential abundance (*P* < 0.05) in the subcutaneous adipose tissue of 110-day-old fetuses and 90-day-old fetuses (Fig. 2), and 52 unique proteins were identified by MS/MS (Additional file 4). Irrespective of age, a total of 75 protein spots differed in abundance (*P* < 0.05) between fetus genotypes; among them, 24 unique proteins were identified. An interaction effect between gestational age and fetus genotype was observed for 76 protein spots corresponding to 13 unique proteins.

### Adipose proteins affected by advancing gestation

The adipose tissue proteins whose abundance was modified with advancing gestation could be clustered into 10 biological processes and 3 main functions (Table 1). The abundance ratios of the protein spots (d 110 vs. d 90) are detailed in Additional file 5. The most apparent effect of advancing gestation was observed on proteins associated with energy-related pathways, lipid binding and the cytoskeleton. Among them, several proteins participating in hexose metabolic process [aldolase (ALDOC), lactate dehydrogenase (LDHB), aldo-keto reductase (AKR1B1), galactose mutarotase (GALM), galactokinase (GALK1)], pyruvate metabolism [pyruvate dehydrogenase (PDHB), malate dehydrogenase (MDH1)] and oxidation reduction [acyl-CoA dehydrogenase (ACADS), prostaglandin reductase (PTGR2), aldehyde dehydrogenase (ALDH9A1), periredoxins (PRDX1, PRDX6)] were more abundant in the subcutaneous adipose tissue of 110-day-old fetuses than in that of 90-day-old fetuses. The ATP synthetase subunit ATP5B was the only protein in these energy-related processes with a decreased abundance between d 90 and d 110. With advancing gestation, there was an increase in the abundance of fatty acid binding proteins (FABP3, FABP4), annexin A4 (ANXA4), chaperonin heat-shock proteins (HSPD1, HSPA1B), non-sarcomeric myosin regulatory light chains (MYL12A, MYL12B) and gelsolin (GSN). Conversely, the abundance of proteins involved in cytoskeleton organization such as cofilin (CFL1), fascin-1 (FSCN1), vinculin (VCL), tropomyosin-3 (TPM3) and actin itself (ACTB) declined with advancing gestation. Other proteins corresponding to microtubule-based processes such as stathmin (STMN1), tubulins (TUBA1B, TUBB) and desmin (DES) also had reduced abundance at d 110. The abundance of Rho GDP-dissociation inhibitor alpha (ARHGDIA) was reduced for 110-day-old fetuses compared with 90-day-old fetuses. Finally, apolipoprotein A1 (APOA1) and vimentin (VIM) were two proteins identified to have proteoforms that varied according to age of development. Additionally, 8 proteins that were not clustered in significantly enriched functional pathways were also found to be differentially expressed between d 90 and d 110; detailed information can be found in Additional file 5.

**Table 1:** Main biological processes and functional categories of proteins in subcutaneous adipose tissue of pig fetuses as affected by age of development

Functional annotation^a^	*P*-value	Enrich.	Identified proteins^b^
Cluster 1 (E score = 3.70)			d 110 ≥ d 90:
Pyruvate metabolism	< 0.001	20.5	LDHB, MDH1, PDHB, AKR1B1,
Oxidation reduction	< 0.001	6.2	ACADS, PTGR2, ALDH9A1, PRDX1, PRDX6
Cluster 2 (E score = 2.55)			d 110 ≥ d 90:
Glycolysis	< 0.001	13.7	LDHB, MDH1, PDHB, ALDOC,
Hexose process	< 0.001	9.0	GALM, GALK1, ALDH9A1
Generation of precursor metabolites and energy	0.02	4.6	d 90 ≥ d 110: ATP5B
Cluster 3 (E score = 2.46)			d 110 ≥ d 90:
Actin binding	0.003	7.8	GSN d 90 ≥ d 110: CFL1, VCL, FSCN1, TPM3
Cluster 4 (E score = 2.11)			d 110 ≥ d 90: FABP3, FABP4, APOA1 d 90 ≥ d 110: VCL, APOA1
Lipid binding	< 0.001	85.4	
Cluster 5 (E score = 2.01)			d 110 ≥ d 90: HSPD1, HSPA1B d 90 ≥ d 110: HSPA8, CLIC1
Response to protein stimulus	0.005	10.8	
Molecular chaperone	< 0.001	88.8	
Cluster 6 (E score = 1.67)			d 110 ≥ d 90: GSN, MYL12A, MYL12B, CRKL d 90 ≥ d 110: CFL1, ACTB, VCL
Regulation of actin cytoskeleton	0.001	5.3	
Cluster 7 (E score = 1.59)			d 110 ≥ d 90: HSPD1, HSPA1B, PHB, PRDX1, ANXA4 d 90 ≥ d 110: CFL1, LGALS1, ARHGDIA
Regulation of apoptosis	0.020	2.9	
Cluster 8 (E score = 1.47)			d 110 ≥ d 90: GSN
Cytoskeleton organization	0.015	4.0	
Actin filament organization	0.02	12.0	d 90 ≥ d 110: TUBA1B, TUBB, CFL1, FSCN1, TPM3, DES, STMN1
Cluster 9 (E score = 1.46)			d 110 ≥ d 90: CRKL, APOA1 d 90 ≥ d 110: ARHGDIA, CFL1, APOA1
Ras protein signal transduction	0.005	10.9	
Rho protein signal transduction	0.007	22.8	
Cluster 10 (E score = 1.45)			d 110 ≥ d 90: ALDOC, ACADS, FABP3, FABP4 d 90 ≥ d 110: AHSG, CFL1, SERPINA1
Response to endogenous stimulus	0.002	5.0	
Response to hormone stimulus	0.03	3.9	
Cluster 11 (E score = 1.41)			d 110 ≥ d 90: PRDX1, PRDX6 d 90 ≥ d 110: PDIA6
Cell redox homeostasis	0.020	13.7	
Cluster 12 (E score = 1.18)			d 110 ≥ d 90: TPM3, APOA1, VIM d 90 ≥ d 110: ATP5B, CFL1, ACTB, VCL, ARHGDIA, APOA1, VIM
Cell motion	0.001	4.8	
Cluster 13 (E score = 1.10)			d 110 ≥ d 90: CLEC3B, APOA1 d 90 ≥ d 110: AFP, AHSG, SERPINA1, APOA1
Plasma	< 0.001	20.6	

^a^The E score of the cluster was measured by minus log transformation of the geometric mean of the modified Fisher Exact EASE Scores of all annotation terms that belong to this cluster, and it was intended to order the relative importance of the different clusters. The fold enrichments (enrich.) of specific individual annotation terms were also indicated for the most biologically-informative terms within each cluster

^b^Lists of regulated proteins participating to each cluster were indicated. d 110 > d 90: proteins having a higher abundance in subcutaneous adipose tissue of fetuses at 110 d of gestation than at 90 d of gestation. d 90 > d 110: proteins having a higher abundance in subcutaneous adipose tissue of fetuses at 90 d of gestation than at 110 d of gestation. APOA1 and VIM were represented by spots with an opposite regulation by age of gestation

### Adipose proteins affected by fetal genotype

Most of the proteins whose abundance was affected by fetal genotype (Table 2) participated in biological pathways that were shown to differ between the two gestational stages. Supporting information is provided in Additional file 6. In particular, proteins responding to nutrient levels such as aldolase (ALDOC), nucleoside diphosphate kinase (NME1) and fatty acid binding proteins (FABP3, FABP4) were more abundant in MeiS fetuses than in LW fetuses. Similarly, proteins involved in the response to oxidative stress such as the heat shock protein HSPA1B, serum albumin (ALB) and the chloride intracellular channel CLIC1 were more abundant in MeiS fetuses than in LW fetuses. Other than CLIC1, the abundance levels of these proteins in F1_MeiS fetuses were close to those measured in MeiS littermates, whereas in F1_LW fetuses, they were generally intermediate between those of pure LW and pure MeiS fetuses. As a consequence, FABP3, ALDOC, NME1 and TF in adipose tissue were more abundant in crossbred fetuses gestated in a MeiS uterus than in crossbred fetuses gestated in an LW uterus. In the category of proteins regulating cytoskeleton organization, gelsolin (GSN) and spectrin (SPTAN1) were more highly expressed in MeiS fetuses than in LW fetuses. By contrast, the abundance of other proteins participating in cytoskeleton organization, such as capping actin protein of muscle Z-line beta subunit (CAPZB) and myosin light chains 6 (MYL6) and 12A (MYL12A) were lower in MeiS fetuses than in LW fetuses; the abundance of these proteins in F1 fetuses was close to the levels measured in LW fetuses. In addition, ARHGDIA was less abundant when fetuses grew in a MeiS uterus (i.e., MeiS and F1_MeiS) than when fetuses grew in an LW uterus (i.e., LW and F1_LW). Similarly, proteins with an anti-oxidative capacity (PRDX6) and participating in apoptotic cell clearance such as protein disulfide isomerases (PDIA3 and PDIA6) had a lower abundance in MeiS fetuses than in LW fetuses. An intracellular thiol protease inhibitor identified as type 1 cystatin (CSTB) was also less highly expressed in MeiS than in LW fetuses. The abundance levels of these redox proteins in F1 fetuses were intermediate between those found in the adipose tissue of MeiS and LW fetuses.

**Table 2:** Main biological processes and functional categories of proteins in subcutaneous adipose tissue of pig fetuses as affected by genotype

Functional annotation^a^	*P*-value	Enrich.	Identified proteins^b^
Cluster 1 (E score = 2.44)			MeiS > LW: SPTAN1, GSN LW ≥ MeiS: ARHGDIA,MYL6, MYL12A, VIM, APOA1, CAPZB F1_LW ≥ F1_MeiS: ARHGDIA, APOA1
Negative regulation of cellular component organization	< 0.001	21.6	
Actin capping Regulation of actin filament-based process	< 0.001 0.008	126 20.0	
Cluster 2 (E score = 2.17)			MeiS > LW: FABP3, FABP4, TF, CLIC1 LW ≥ MeiS: APOA1 F1_MeiS > F1_LW: FABP3, TF F1_LW ≥ F1_MeiS: APOA1
Lipid binding PPAR signaling pathway	0.02 0.01	133.0 17.0	
Cluster 3 (E score = 1.96)			MeiS > LW: HSPA1B, ALB, NME1 LW ≥ MeiS: ARHGDIA, PDIA3, CSTB F1_MeiS > F1_LW: ALDOC, FABP3, TF, NME1 F1_LW ≥ F1_MeiS: ARHGDIA
Regulation of apoptosis Regulation of programmed cell death	0.007 0.007	4.5 4.5	
Cluster 4 (E score = 1.74)			LW ≥ MeiS: PRDX6, PDIA6, PDIA3
Cell redox homeostasis	0.004	29.3	
Cluster 5 (E score = 1.40)			MeiS > LW: TF, ALB LW ≥ MeiS: APOA1 F1_MeiS > F1_LW: TF F1_LW ≥ F1_MeiS: APOA1
Plasma	0.004	25.8	
Cluster 6 (E score = 1.23)			MeiS > LW: ALDOC, NME1, FABP3, FABP4, CLIC1, TF, ALB, HSPA1B, GSN F1_MeiS > F1_LW: ALDOC, FABP3, TF, NME1
Response to nutrient levels Response to extracellular stimulus	0.04 0.04	9.4 8.4	

^a^The E score of the cluster was measured by minus log transformation of the geometric mean of the modified Fisher Exact EASE Scores of all annotation terms that belong to this cluster, and it was intended to order the relative importance of the different clusters. The fold enrichments (enrich.) of specific individual annotation terms were also indicated for the most biologically-informative terms within each cluster

^b^Lists of regulated proteins participating to each cluster were indicated. MeiS > LW: proteins having a higher abundance in pure Meishan than in pure Large White genotypes. LW > MeiS: proteins having a higher abundance in pure Large White than in pure Meishan genotypes. F1_MeiS > F1_LW: proteins having a higher abundance in crossbred (F1) fetuses gestated by MeiS sows when compared with those gestated by Large White sows. F1_LW > F1_MeiS: proteins having a higher abundance in crossbred (F1) fetuses gestated by Large White sows when compared with those gestated by Meishan sows

### Interaction effects between developmental age and fetus genotype

**Fig. 3:**
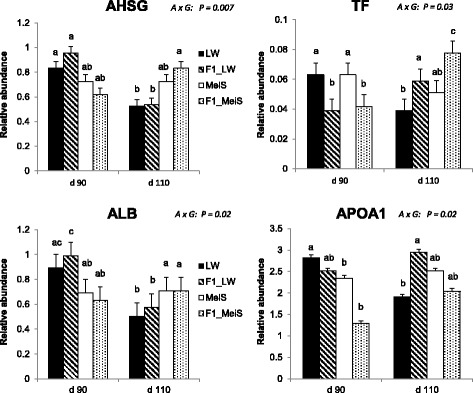
Differences in abundance of secreted adipose proteins in purebred and crossbred Large White or Meishan fetuses gestated by sows of either breed at d 90 or d 110 of gestation. Four fetal genotypes were analyzed (*n* = 6 per age and per genotype). LW and F1_LW were from LW sows, whereas MeiS and F1_MeiS were from MeiS sows. The abundance of a given protein spot was expressed relative to an internal standard; values were log transformed. When multiple spots corresponded to the same identified protein, the mean abundance was used to produce the bar charts. Analysis of variance was used to determine whether fetus genotype altered the time-course changes in the abundance of the proteins in dorsal subcutaneous adipose tissues between d 90 and d 110 of gestation. The *P*-value for the interaction effect between age and genotype (A × G) is indicated for selected adipose proteins. AHSG: alpha-2-HS glycoprotein; ALB: albumin; APOA1: apolipoprotein A1; TF: transferrin Least squares means sharing a common superscript letter did not significantly differ (*P* > 0.05)

Proteins affected by an interaction (*P* < 0.05) between developmental age and fetal genotype were involved in two main pathways: one is associated with responses to organic substances, and the other is related to the acute-phase response (Table 3). The complete list of proteins is given in Additional file 7. Various adipose proteins known to be secreted were identified. In particular, fragments of alpha-2-HS-glycoprotein (AHSG) as well as albumin (ALB) exhibited reduced abundance with advancing gestation in LW and F1_LW fetuses, whereas the abundance of these proteins remained almost constant between the two ages in the adipose tissue of MeiS and F1_MeiS fetuses (Fig. 3). Apolipoprotein A1 (APO1) was another protein with a lower abundance at d 110 than at d 90 in LW fetuses, whereas its abundance was stable (F1_LW and MeiS) or increased (F1_MeiS) with advancing gestation in the three other genotypes. The abundance of transferrin (TF) was significantly lower in F1 fetuses than in their purebred littermates at d 90 but was the highest in the crossbred genotypes at d 110. Among other identified proteins, isocitrate dehydrogenase (IDH1) was also oppositely regulated in MeiS and LW fetuses during the last month of gestation. Indeed, this protein was less abundant in 110-d-old fetuses than in 90-day-old fetuses of the LW genotype, but it was higher in 110-day-old than in 90-day-old MeiS fetuses (Additional file 7: Table S7). As a consequence, IDH1 was approximately twofold more abundant in adipose tissue of pure MeiS fetuses than in that of pure LW fetuses at d 110 of gestation.

**Table 3:** Main functional categories of proteins affected by an interaction between genotype and developmental age effects in subcutaneous adipose tissue of pig fetuses

Functional annotation^a^	*P*-value	Enrich*.*	Proteins
Cluster 1 (E score = 2.75)			AHSG, TF, GSN,
Response to organic substance	< 0.001	9.4	SERPINA1, PEPB1,
Response to hormone stimulus	0.003	12.3	IDH1
Cluster 2 (E score = 2.48)			
Acute phase response	< 0.001	84.6	AHSG, SERPINA1, TF
Cluster 3 (E score = 1.90)			
Negative regulation of signal transduction	0.013	15.3	AHSG, PEPB1, CALR
Cluster 4 (E score = 1.65)			APOA1, CALR, TF
Chemical homeostasis	0.007	8.8	HSP90B1
Cluster 5 (E score = 1.47)			AHSG, PEPB1, CALR,
Macromolecular complex assembly	0.015	6.8	GSN
Cluster 6 (E score = 1.38)			
Maintenance of location	0.01	52.8	ALB, PEPB1, CALR
Regulation of apoptosis	0.10	4.2	

^a^The E score of the cluster was measured by minus log transformation of the geometric mean of the modified Fisher Exact EASE Scores of all annotation terms that belong to this cluster, and it was intended to order the relative importance of the different clusters. The fold enrichments (enrich.) of specific individual annotation terms were also indicated for the most biologically-informative terms within each cluster

### Expression levels of target genes

**Fig. 4:**
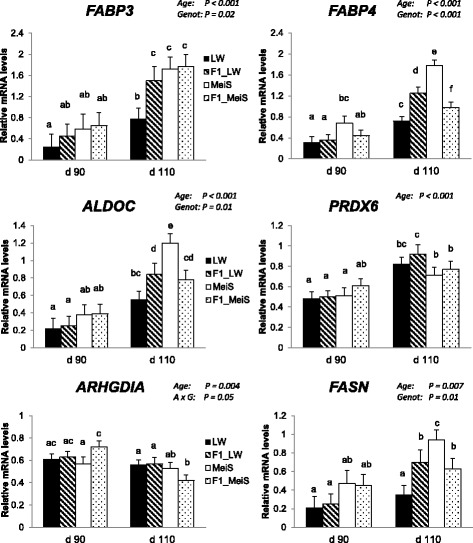
Expression levels of genes encoding metabolically active proteins in subcutaneous adipose tissue of purebred and crossbred Large White or Meishan fetuses gestated by sows of either breed. Subcutaneous adipose tissues sampled from 4 fetal genotypes on d 90 or d 110 of development were analyzed (*n* = 6 per age and per genotype). LW and F1_LW were from LW sows, whereas Meishan (MeiS) and F1_MeiS were from MeiS sows. The mRNA levels of target genes in dorsal subcutaneous adipose tissue were measured by qPCR. Analysis of variance was used to determine the main effects of developmental age and fetal genotype and their interaction effect (age × genotype) on expression levels of the target genes. Values were expressed as mRNA levels of the target gene relative to mRNA levels of housekeeping genes (arbitrary units). Least squares means sharing a common superscript letter did not significantly differ (*P* > 0.05)

The expression levels of different genes were examined by qPCR to support and extend the proteomics data. As expected, both developmental age and fetal genotype affected the expression levels of the adipocyte-type (*FABP4*) and heart/muscle-type (*FAPB3*) fatty acid binding proteins as well as that of aldolase (*ALDOC*). Differences between pure LW and pure MeiS fetuses were clearly observed at d 110, with the lowest expression level in LW fetuses (Fig. 4). Similarly, the mRNA levels of *PRDX6* increased with advancing gestation; however, there was no significant difference among the four fetal genotypes. Conversely, a decrease in *ARHGDIA* mRNA level was observed with advancing age (*P* < 0.001), and this decrease was much more accentuated in F1_MeiS than in the 3 other genotypes.

**Fig. 5:**
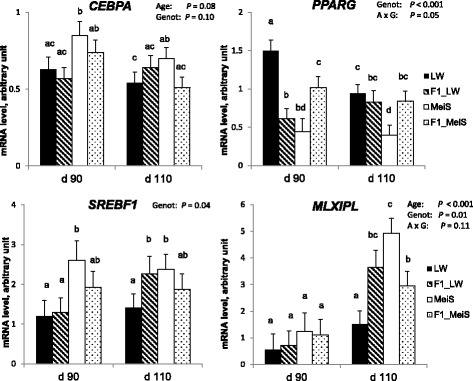
Expression levels of transcriptional regulators in subcutaneous adipose tissue of purebred and crossbred Large White or Meishan fetuses gestated by sows of either breed. Subcutaneous adipose tissues sampled from 4 fetal genotypes at d 90 or d 110 of development were analyzed (*n* = 6 per age and per genotype). LW and F1_LW were from LW sows, whereas MeiS and F1_MeiS were from MeiS sows. The mRNA levels of transcriptional regulators in dorsal subcutaneous adipose tissue were measured by qPCR. Analysis of variance was used to determine the main effects of developmental age, fetal genotype and their interaction (A × G) on the expression levels of the target genes. Values were expressed as mRNA levels of the target gene relative to mRNA levels of housekeeping genes (arbitrary units). Least squares means sharing a common superscript letter did not significantly differ (*P* > 0.05)

To extend this analysis, we also studied genes known to play a significant role in adipogenesis and lipogenesis. The expression level of *FASN* increased (*P* < 0.01) with gestational age (Fig. 4). At d 110 of gestation, *FASN* expression was higher in MeiS fetuses than in LW fetuses and did not differ between F1 crossbred genotypes. Similarly, *MLXIPL* was up-regulated (*P* < 0.001) by advancing gestation (Fig. 5). At d 110, its expression was higher in the adipose tissue of pure MeiS fetuses than in that of pure LW fetuses (*P* < 0.05) and intermediate in the two types of F1 fetuses. *SREBF1* expression was not affected by age during the studied period, but it exhibited a greater level of expression in pure MeiS fetuses than in pure LW fetuses at both time points (*P* < 0.05); its expression level was almost similar in F1_LW and F1_MeiS fetuses. The expression level of *CEBPA*, a gene involved in cell cycle regulation and homeostasis, tended to decrease (*P* = 0.08) between d 90 and d 110 of gestation; the highest levels were found at d 90 in the adipose tissue of pure MeiS fetuses and, to a lesser extent, of crossbred F1_MeiS. Finally, *PPARG* was significantly down-regulated during the last month of gestation in the adipose tissue of pure LW fetuses only. At both time points, it was more highly expressed in pure LW fetuses and less highly expressed in pure MeiS fetuses.

### Plasma concentrations of nutrients and IGF-I

**Fig. 6:**
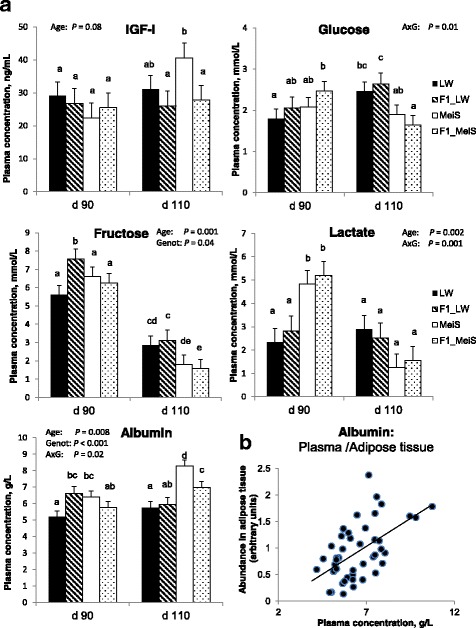
Plasma concentrations of IGF-I and nutrients in purebred and crossbred Large White or Meishan fetuses gestated by sows of either breed. Blood samples taken from 4 fetal genotypes at d 90 or d 110 of development were analyzed (*n* = 6 per age and per genotype). LW and F1_LW were from LW sows, whereas MeiS and F1_MeiS were from MeiS sows. Analysis of variance was used to determine the main effects of developmental age and fetal genotype and their interaction effect (A x G) on plasma concentrations of IGF-I, glucose, fructose, lactate and albumin (**a**). The albumin concentration in plasma was plotted against the abundance of albumin in the adipose tissue of the same fetuses (**b**)

Plasma IGF-I concentration increased during late gestation in pure MeiS fetuses, whereas it remained almost stable in the three other genotypes (Fig. 6a); it was the highest in pure MeiS fetuses at d 110. There was an interaction effect of developmental age and fetal genotype on the circulating concentrations of the studied nutrients (except fructose). Plasma glucose concentration increased from d 90 to d 110 in pure and crossbred fetuses gestated by the LW sows but remained stable in pure MeiS and decreased during this period in F1_MeiS fetuses. At d 110, plasma glucose tended to be lower (*P* = 0.10) in fetuses gestated by MeiS sow than in fetuses from LW sows. Plasma fructose decreased (*P* < 0.001) from d 90 to d 110 in all genotypes. The circulating concentration of fructose was the highest in F1 fetuses gestated by LW sows at both ages, and it was the lowest in pure and crossbred fetuses gestated by MeiS sows at d 110. Blood fructose was about threefold higher than glucose in fetuses at d 90, whereas differences were no longer observed at d 110. The circulating concentration of lactate remained almost stable in fetuses gestated by LW sows but dramatically decreased (*P* < 0.05) from d 90 to d 110 in fetuses gestated by MeiS sows. The plasma concentration of albumin increased (*P* < 0.01) from d 90 to d 110 in MeiS and F1_MeiS but remained almost stable during this period in LW and F1_LW fetuses. The plasma albumin concentration was greater in MeiS fetuses than in LW fetuses at both ages. At d 110, it was also greater in crossbred fetuses that grew in MeiS uteri (F1_MeiS) than those developing in LW uteri (F1_LW). There was a positive correlation (*r* = 0.55) between albumin concentration in plasma and albumin abundance in fetal adipose tissue (Fig. 6b).

## Discussion

Approximately 60% of uterine energy deposition in sows occurs during the last 30 d of gestation, [25] and the body weight of pig fetuses almost doubles (present results; [26]) during this period. Previous studies have proposed d 90 of gestation as the midway point of adipose tissue development in pig fetuses [27]. The present study brings new clues that allow a better understanding of the maturation of pig adipose tissue during the last month of gestation.

### Identification of proteomic changes in subcutaneous adipose tissue of pig fetuses due to advancing gestation

Proteins involved in 10 biological pathways underwent pronounced changes from d 90 to d 110 after conception. Specifically, they were associated with metabolism, the cytoskeleton, redox, chaperones, Rho protein signal transduction regulation, apoptosis and others; these pathways correspond to the categories of proteins found to be regulated during adipocyte differentiation in cell culture models [28–30]. Most of these proteins that increase in abundance with advancing gestation are known to play important roles in energy metabolic processes. This suggests an increased capacity for carbohydrate handling and oxidation in the intact adipose tissue of pig fetuses from d 90 to d 110, which is in agreement with previous histochemical studies [31–33] and molecular analyses [23–34] showing an increase in the activity of lipogenic enzymes and a decrease in ATPase activity in pig adipose tissue with advancing gestation. During the last month of gestation, ANXA4, a protein playing roles in carbohydrate recognition [35], and gelsolin (GSN), a calcium-regulated protein having specific roles in promoting lipogenic genes in 3 T3-L1 adipocyte cells [36], increase in abundance, which also supports this view. These changes associated with the marked increase in expression (both at the mRNA and protein levels) of FASN, the key enzyme catalyzing fatty acid synthesis, and FABP4, the adipocyte-type fatty acid binding protein, may explain the doubling in adipose tissue triglyceride content during this period. Conversely, we showed a decreased abundance with advancing gestation in different fragments of cytoskeletal proteins, notably, several related to tubulin (such as α- and β-tubulins and stathmin) and actin (such as TPM3, a protein that provides stability to actin filaments, and β-actin itself). These changes are likely associated with the programmed morphological changes that occurr during adipogenic differentiation [28–30, 37, 38].

### Genotype-associated differences in the abundance of several metabolic proteins suggest better anabolic function of adipose tissue in pure MeiS fetuses

When analyzed a few days before birth, the pure MeiS fetuses exhibited the highest triglycerides content in subcutaneous adipose tissue, although they have lower body weight than pure LW or crossbred F1 fetuses. This finding contrasts with the positive relationship usually reported between body fat mass and birth weight when these traits were analyzed within European breeds [8]. Various proteins involved in lipid metabolism (ALDOC, FABP3, FABP4, and GSN) had a greater abundance in the adipose tissue of MeiS fetuses than in that of LW fetuses. A greater abundance in IDH1, an enzyme involved in cytosolic NADPH production as an essential cofactor for lipogenesis, was also found in the MeiS fetuses at d 110 of gestation. At the mRNA level, we showed that *FASN*, the key lipogenic enzyme; *MLXIPL*, a pivotal transcriptional mediator of glucose-related stimulation of lipogenic genes in porcine primary adipocytes [39]; and *SREBF1*, another classically recognized gene regulator controlling the expression of genes participating in the use of glucose for anabolic purposes [40], were more highly expressed in the adipose tissue of pure MeiS than in that of pure LW fetuses. Altogether, these data strongly argue that the adipose tissue of pure MeiS fetuses has superior metabolic function. This may explain why Meishan neonates exhibited a greater number of enlarged adipose cells than did the Large White neonates [11]. Because contrasting time-course changes between pure MeiS and LW fetuses were observed in the expression levels of *PPARG* and *CEBPA,* two transcriptional regulators of adipogenesis, time points earlier in gestation may be considered in further studies to clarify breed-associated differences in adipose cell development. Finally, three redox proteins (PRDX6, PDIA3, and PDIA6) were less abundant in MeiS than in LW fetuses at the two gestational ages. Up-regulation of redox proteins has been reported to occur during adipocyte development, when cells must cope with increased production of reactive oxygen species (ROS) [41], and two proteins of the peroxiredoxin family (PRDX1, PRDX6) were also found to be more abundant in pig adipose tissue at d 110 than at d 90 of gestation in the present study. Taken together, these data suggest that ROS generation was lower and/or that ROS scavenging was more effective in MeiS adipose tissue. The increased abundance of CLIC1, a chloride intracellular channel whose knockdown in cells led to increased sensitivity to hydrogen peroxide [42], was another element supporting this assumption.

### Differences in the abundance of adipose proteins with regulatory roles in cellular growth were revealed between fetal genotypes

At the two examined time points of gestation, proteins participating in the regulation of cytoskeleton organization and filament dynamics (MYL6, MYL12A, VIM and CAPZB) were decreased in abundance in the subcutaneous adipose tissue of pure MeiS fetuses. These differences may reflect the lower number of connective tissue fibers reported to exist in the interstitial space of the adipose tissue in neonates from obese breeds compared with lean breeds [43]. Because proteins included in this pathway exhibited decreased abundance with advancing gestation, these data further argue that the adipose tissue of MeiS fetuses just before birth is more mature than that of LW fetuses or hybrids. During stem cell lineage commitment, several proteins are known to activate a number of downstream effectors regulating cytoskeletal organization, thereby exerting effects on cellular growth. Among them, Rho proteins including Rho GDP-dissociation inhibitor alpha (ARHGDIA) are important actors in the regulation of cell growth [44, 45]. In particular, the down-regulation of ARHGDIA may promote the proliferation, cell cycle progression and migration of different cell types [46]. Moreover, Rho GTPases are known to regulate the phosphorylation of myosin regulatory light chains [47]. In the present study, ARHGDIA protein and the myosin light chain elements MYL12A and MYL6 were less abundant in the adipose tissue of MeiS fetuses. In addition, we showed that cystatin B (CSTB), a protein encoded by a gene whose down-regulation increased cell viability and decreased apoptosis [48], had a lower abundance, whereas spectrin (SPTAN1), a gene involved in tumorigenesis [49], was more abundant in the adipose tissue of pure MeiS fetuses than in that of pure LW fetuses at both gestational time points. Altogether, these protein changes suggested that the regulation of the cell cycle in adipose tissue may be different during the last month of gestation in purebred fetuses of fat vs. lean breeds. In support of this interpretation, preadipocytes have been reported to have an elevated proliferation rate in MeiS compared with LW primary cell cultures [50]. In MeiS fetuses, the sharp increase from d 90 to d 110 in the circulating concentration of IGF-I, a member of the larger family of insulin-related growth factors, may have promoted preadipocyte proliferation [51] in this genotype.

### Maternal influences were important factors explaining differences in the maturity grade of adipose tissue between pig fetuses

Comparing the relative abundance of adipose proteins between pure and crossbred fetuses may reveal new, relevant information regarding the main effects of fetal genetics and uterine environment on the mechanisms involved in adipose tissue maturation. In crossbred fetuses gestated by MeiS sows, the abundance levels of metabolically active proteins known to be responsive to nutrient levels were close to those found in their pure MeiS littermates, whereas in F1_LW fetuses, they were generally intermediate between the levels in pure LW and pure MeiS fetuses. In particular, FABP3, ALDOC, NME1 and TF in adipose tissue were more abundant in crossbred fetuses that developed in a MeiS uterus than in those that developed in an LW uterus. This suggests that uterine influences are important for the metabolic function of adipose tissue during gestation. Differences in placental and endometrial vascularity between MeiS and LW sows during the late stages of gestation may be involved. Indeed, the vascular density of the MeiS placenta increases by about one-third between d 90 and d 110 of gestation while remaining almost constant in LW dams [52]. It is also recognized that uterine type determines conceptus size, whereas conceptus genotype controls placental efficiency [52]. In the current study, circulating concentrations of glucose and fructose, two energy-yielding nutrients, were lower in the arterial blood of 110-day-old pure and crossbred fetuses gestated by MeiS sows than in that of fetuses gestated by LW sows. Time-dependent decreases in circulating glucose and lactate concentrations were also observed in fetuses gestated by MeiS sows, whereas plasma glucose concentration increased with advancing gestation in fetuses gestated by LW sows (present results) and LW × Landrace sows [23]. This suggests that circulating nutrient concentrations might be less important than blood flow for adipose tissue metabolism. Conversely, the abundance of proteins participating in cytoskeleton organization (except ARHGDIA) in the adipose tissue of F1 fetuses was close to that measured in pure LW fetuses. The lack of differences in the abundance of these proteins between the two types of crossbred fetuses suggests that parental genes are more influential than the uterine environment in the regulation of adipose cell growth.

### Adipose secreted proteins may be indicative of accelerated maturation during gestation

Possible indicators of accelerated maturation of adipose tissue in fetuses should be sought among the few identified proteins that show different time courses depending on the fetal genotype. This interaction between age and genotype especially affected certain secreted proteins, namely, albumin, transferrin and fetuin-A, that were previously identified in culture media conditioned by porcine stroma-vascular preadipose cells [16] and serum from fetal and neonatal pigs [53]. In the adipose tissue of pure and crossbred fetuses gestated by LW sows, the abundance of fetuin-A (the protein encoded by the gene *AHSG*) reduced over the course of gestation. This observation is consistent with the time-course changes reported for serum fetuin-A from fetal life to neonatal life in white-type pigs [53]. Conversely, adipose tissue fetuin-A remained almost constant in fetuses gestated by MeiS sows. Given that the mRNA and protein levels of AHSG/fetuin-A increased during differentiation of 3T3-L1 murine adipocytes [54], this was further evidence to support the hypothesis of accelerated adipose tissue maturation in the offspring of MeiS sows during late gestation. Fetuin-A mediates the cross-talk between liver and adipose tissue, and changes in fetuin-A abundance are associated with deficient fetal growth and complications in later life [55]. Its precise role in adipose tissue, however, remains to be clarified. Another adipose tissue protein showing an opposite time-course between pure LW and MeiS fetuses was albumin. In mice, plasma albumin is taken up by white adipose tissue at an almost constant rate from fetal to early postnatal life [56]. Moreover, the albumin concentration in the plasma of pig fetuses increases during late gestation (present results; [23]) but is lower in LW fetuses than in MeiS fetuses at d 110. Similar to the present findings, the serum concentration of albumin was found to be lower in pig neonates from lean breeds than in those from obese breeds [12, 13] and lower in neonates from highly selected white-type sows than in neonates from less selected sows [7]. Finally, high plasma albumin concentration has been suggested to be indicative of advanced development and increased physiological maturity in pig fetuses [57]. Importantly, albumin may regulate fatty acid uptake by the tissue [58], such that inter-individual variations in adipose tissue albumin content could be important for early survival when piglets feed on colostrum and milk.

## Conclusion

During the last three weeks of gestation, increased expression of metabolic proteins with roles in energy generation and lipid binding together with reduced expression of proteins involved in cytoskeleton organization contributed to the rapid development of pig adipose tissue. During this period, differences between fetus genotypes in the abundance of adipose proteins involved in energy metabolism likely contributed to the higher maturity grade of adipose tissue of fetuses gestated by Meishan sows. These proteins may be regulated by differences in maternal environment, such as placental efficiency. Altogether, however, the number of proteins that were differentially regulated by fetus genotype was one-third the number regulated by gestational age. This confirmed that although some cellular and metabolic differences can be observed between obese and lean pig breeds at d 110 of gestation [32], maternal obesity has less visible influence on adipose tissue during gestation than during the postnatal period [59]. Accelerated developmental changes in adipose tissue during the fetal period may program the greater fat accretion observed in the Meishan breed during postnatal life.

## Additional files

Additional file 1: Running plan for the 48 samples in Differential Gel Electrophoresis. (DOCX 29 kb)

Additional file 2: Representative two dimensional gels of fetal adipose proteins stained by silver nitrate. The first image corresponds to subcutaneous adipose tissue d 90, the second one at d 110 of gestation for pure Large White fetuses. (DOCX 1257 kb)

Additional file 3: Primers for target gene expression by qPCR. (DOCX 30 kb)

Additional file 4: Mass parameters for protein identities. (XLS 474 kb)

Additional file 5: Proteins showing a differential abundance in adipose tissue with developmental age. (DOCX 42 kb)

Additional file 6: Proteins showing a differential abundance in adipose tissue according to fetus genotype. (DOCX 96 kb)

Additional file 7: Proteins in adipose tissue affected by age in a different manner according to fetus genotype. (DOCX 32 kb)

## Abbreviations

2D–DIGE: Two-dimension differential gel electrophoresis
ACADS: Acyl-CoA dehydrogenase
ACTB: Actin beta
AHSG: Alpha2-HS glycoprotein
AKR1B1: Aldo-keto reductase family 1, member B1
ALB: Albumin
ALDH9A1: Aldehyde dehydrogenase 9 family member A1
ALDOC: Aldolase-C
APOA1: Apolipoprotein-A1
ARHGDIA: Rho GDP-dissociation inhibitor 1
ATP5B: ATP synthase subunit beta
CAPZB: F-actin-capping protein subunit beta
CFL1: Cofilin
CLIC1: Chloride intracellular channel 1
CTSB: Cystatin
d 110: 110 days of gestation
d 90: 90 days of gestation
DES: Desmin
FABP3: Fatty acid binding protein heart/muscle type
FABP4: Fatty acid binding protein adipocyte type
FSCN1: Fascin actin-bundling protein 1
GSN: Gelsolin
HSPA1B: Heat shock protein family A (Hsp70) member 1B
HSPD1: Heat shock protein family D (Hsp60)
IDH1: Isocitrate dehydrogenase
LDHB: Lactate dehydrogenase B
LW: Large White
MDH1: Malate dehydrogenase 1
MeiS: Meishan
MS: Mass spectrometry
MYL12A: Myosin light chain 12A
MYL6: Myosin light chain 6
PDHB: Pyruvate dehydrogenase
PDIA3: Protein disulfide-isomerase A3
PDIA6: Protein disulfide-isomerase A3
PRDX1: Peroxiredoxin-1
PRDX6: Peroxiredoxin-6
PTGR2: Prostaglandin reductase 2
ROS: Reactive oxygen species
SPTAN1: Spectrin alpha-chain
STMN1: Stathmin 1
TF: Transferrin
TPM3: Tropomyosin 3
TUBA1B: Tubulin alpha-1B
TUBB: Tubulin beta class 1
VCL: Vinculin
VIM: Vimentin
