# Supplementary material for: Proteomic analysis of adipose tissue during the last weeks of gestation in pure and crossbred Large White or Meishan fetuses gestated by sows of either breed
Source: J Anim Sci Biotechnol. 2018 Apr 3;9:28. doi: 10.1186/s40104-018-0244-2 (PMC5881184; doi:10.1186/s40104-018-0244-2)
Supplement: Supplementary file 1 — Running plan for the 48 samples in Differential Gel Electrophoresis. (DOCX 29 kb) [file 40104_2018_244_MOESM1_ESM.docx]

Additional File 1. Running plan for the 48 samples in Differential Gel Electrophoresis

| Series | Gel | Cye 3 | Cye 5 | Cye 2 |
| --- | --- | --- | --- | --- |
| 1 | 1 | LW 90-1 | F1_MeiS 90-1 | Standard |
| 1 | 2 | F1_MeiS 110-1 | LW 90-2 | Standard |
| 1 | 3 | LW 110-3 | F1_LW 110-2 | Standard |
| 1 | 4 | F1_MeiS 90-2 | LW 110-4 | Standard |
| 1 | 5 | MS 90-1 | F1_LW 90-3 | Standard |
| 1 | 6 | F1_LW 110-3 | MeiS 90-2 | Standard |
| 2 | 1 | MeiS 110-3 | F1_MeiS 110-4 | Standard |
| 2 | 2 | F1_LW 90-4 | MeiS 110-4 | Standard |
| 2 | 3 | LW 90-5 | F1_MeiS 90-5 | Standard |
| 2 | 4 | LW 110-5 | F1_LW 90-5 | Standard |
| 2 | 5 | F1_MeiS 90-6 | MeiS 90-5 | Standard |
| 2 | 6 | F1_LW 90-6 | MeiS 110-5 | Standard |
| 3 | 1 | LW 90-3 | F1_LW 90-1 | Standard |
| 3 | 2 | F1_LW 110-1 | LW 90-4 | Standard |
| 3 | 3 | LW 110-1 | F1_MeiS 110-2 | Standard |
| 3 | 4 | F1_LW 90-2 | LW 110-2 | Standard |
| 3 | 5 | MeiS 90-3 | F1 MeiS 90-3 | Standard |
| 3 | 6 | F1_MeiS 110-3 | MeiS 90-4 | Standard |
| 4 | 1 | MeiS 110-1 | F1_LW 110-4 | Standard |
| 4 | 2 | F1_MeiS 90-4 | MeiS 110-2 | Standard |
| 4 | 3 | F1_MeiS 110-5 | LW 90-6 | Standard |
| 4 | 4 | F1_LW 110-5 | LW 110-6 | Standard |
| 4 | 5 | MeiS 90-6 | F1_MeiS 110-6 | Standard |
| 4 | 6 | MeiS 110-6 | F1_LW 110-6 | Standard |

Various combinations of samples labelled with Cye 3 or Cye 5 were run with the Cy2-labeled pool in 4 different series of 6 gels each, including dye-swap of labelled extracts to prevent dye-specific protein labelling bias arising from the fluorescence properties of gels at different wavelengths. LW: pure Large White; MeiS: pure Meishan; F1_MeiS: fetus having maternally-derived MeiS genes and paternally-derived LW genes and gestated by a MeiS sow; F1_LW: fetus having maternally-derived LW genes and paternally-derived MeiS genes and gestated by a LW sow. 90: d90 of gestation; 110: d110 of gestation. At each stage of gestation, *n* = 6 fetuses were considered in each fetal genotype.
