# Supplementary material for: Proteomic analysis of adipose tissue during the last weeks of gestation in pure and crossbred Large White or Meishan fetuses gestated by sows of either breed
Source: J Anim Sci Biotechnol. 2018 Apr 3;9:28. doi: 10.1186/s40104-018-0244-2 (PMC5881184; doi:10.1186/s40104-018-0244-2)
Supplement: Supplementary file 2 — Representative two dimensional gels of fetal adipose proteins stained by silver nitrate. The first image corresponds to subcutaneous adipose tissue d 90, the second one at d 110 of gestation for pure Large White fetuses. (DOCX 1257 kb) [file 40104_2018_244_MOESM2_ESM.docx]

Additional file 2 Representative tow dimensional gels of fetal adipose proteins stained by silver nitrate. The first image corresponds to adipose tissue at d90, the second one at d110 of gestation for pure Large White fetuses

pH4 pH7

116

kDa

6

6

116

kDa
