# Supplementary material for: Proteomic analysis of adipose tissue during the last weeks of gestation in pure and crossbred Large White or Meishan fetuses gestated by sows of either breed
Source: J Anim Sci Biotechnol. 2018 Apr 3;9:28. doi: 10.1186/s40104-018-0244-2 (PMC5881184; doi:10.1186/s40104-018-0244-2)
Supplement: Supplementary file 3 — Primers for target gene expression by qPCR. (DOCX 30 kb) [file 40104_2018_244_MOESM3_ESM.docx]

Additional file 3 Primers for target genes

| Gene symbol | Gene name | Accession | Primers |
| --- | --- | --- | --- |
| *ALDOC* | Aldolase-C | NM001243928.1 | Forward: TCCCCTTCGTCCGTACCAT  Reverse: CCAGTCCTTGAGTGGTTGTTTCT |
| *ARHGDIA* | Rho GDP Dissociation Inhibitor Alpha | NM001201384.1 | Forward: GGGTGTGGAATACCGGATAAAG  Reverse: CTTCATGCCCGACACGATCT |
| *CEBPA* | CCAAT/enhancer binding protein alpha | AF103944 | Forward: GTGGACAAGAACAGCAACGA  Reverse: CTCCAGCACCTTCTGTTGAG |
| *FABP3* | Fatty acid binding protein, heart | NM001099931.1 | Forward: CACTTACGAGAAAGAGGCATGA  Reverse: GCTGAGTCCAGGAGTAGCCAATT |
| *FABP4* | Fatty acid binding protein, adipocyte | AJ416020 | Forward: GGAAAGTCAAGAGCACCATAACCT  Reverse: ATTCCACCACCAACTTATCATCTACTATTT |
| *FASN* | Fatty Acid Synthase | NM001099930.1 | Forward: AGCCTAACTCCTCGCTGCAAT  Reverse: TCCTTGGAACCGTCTGTGTTC |
| *HPRT1* | Hypoxanthine phosphoribosyl-transferase | DQ845175 | Forward: TACCTAATCATTATGCCGAGGATTT  Reverse: AGCCGTTCAGTCCTGTCCAT |
| *MLXIPL* | MLX interacting protein like (CHREBP) | ENSSSCT00000008453 | Forward: CGAGGTGGTGATGCGAGAAT  Reverse: TTGCGGAGCCGCTTCTT |
| *PPARG* | Peroxisome proliferator activated receptor gamma | ENSSSCT00000012672 | Forward: ATTCCCGAGAGCTGATCCAA  Reverse: TGGAACCCCGAGGCTTTAT |
| *PPIA* | Peptidylprolyl isomerase A | NM_214353 | Forward: AGCACTGGGGAGAAAGGATT  Reverse: AAAACTGGGAACCGTTTGTG |
| *PRDX6* | Peroxiredoxin-6 | NM214408.1 | Forward: TGCCTGGAGCAAGGATATCAA  Reverse: AGGTCCCGACTCTTATCATCAATG |
| *SREBF1* | Sterol regulatory element binding transcription factor 1 | NM_214157.1 | Forward: CGGACGGCTCACAATGC  Reverse: GCAAGACGGCGGATTTATTC |
