# Supplementary material for: Proteomic analysis of adipose tissue during the last weeks of gestation in pure and crossbred Large White or Meishan fetuses gestated by sows of either breed
Source: J Anim Sci Biotechnol. 2018 Apr 3;9:28. doi: 10.1186/s40104-018-0244-2 (PMC5881184; doi:10.1186/s40104-018-0244-2)
Supplement: Supplementary file 5 — Proteins showing a differential abundance in adipose tissue with developmental age. (DOCX 42 kb) [file 40104_2018_244_MOESM5_ESM.docx]

Additional file 4. Proteins showing a differential abundance in adipose tissue with developmental age

| Spot | Protein name | Derived  HUGO | Mean ratio^1^  110/90 | P value^2^  Age | BH^3^  Age | LW  110/90 | F1_LW  110/90 | MeiS 110/90 | F1_MeiS 110/90 |
| --- | --- | --- | --- | --- | --- | --- | --- | --- | --- |
| 1646 | Abhydrolase domain-containing protein 14B | ABHD14B | 1.26 | 0.006 | 0.026 | 1.36 | 1.00 | 1.59 | 1.17 |
| 1194 | Short-chain specific acyl-CoA dehydrogenase mitochondrial | ACADS | 1.23 | 0.003 | 0.011 | 1.01 | 1.23 | 1.40 | 1.35 |
| 1149 | Actin cytoplasmic 1 | ACTB | -1.31 | 0.030 | 0.091 | -1.28 | -1.13 | -1.53 | -1.36 |
| 712 | Alpha-fetoprotein | AFP | -1.69 | <0.001 | 0.002 | -2.17 | -1.53 | -1.92 | -1.31 |
| 722 |  |  | -1.78 | <0.001 | <0.001 | -2.32 | -1.61 | -2.12 | -1.35 |
| 731 |  |  | -1.69 | <0.001 | 0.003 | -2.00 | -1.69 | -1.56 | -1.56 |
| 732 |  |  | -1.92 | <0.001 | <0.001 | -2.12 | -1.20 | -1.49 | -1.45 |
| 768 |  |  | -1.52 | <0.001 | 0.004 | -2.13 | -1.19 | -1.49 | -1.45 |
| 863 | Alpha-2-HS-glycoprotein (fragment) | AHSG | -1.39 | 0.004 | 0.018 | -2.14 | -1.89 | -1.01 | 1.09 |
| 871 |  |  | -1.21 | 0.030 | 0.084 | -1.51 | -1.69 | 1.05 | 1.14 |
| 921 |  |  | -1.39 | <0.001 | <0.001 | -1.29 | -1.32 | -1.61 | -1.37 |
| 1334 | Aldose reductase | AKR1B1 | 1.62 | <0.001 | 0.002 | 1.14 | 2.11 | 2.34 | 1.32 |
| 985 | Aldehyde dehydrogenase | ALDH9A1 | 2.45 | <0.001 | <0.001 | 1.72 | 2.58 | 2.74 | 3.25 |
| 1191 | Fructose biphosphate aldolase | ALDOC | 1.53 | <0.001 | <0.001 | 1.29 | 1.44 | 1.74 | 1.68 |
| 1193 |  |  | 2.36 | <0.001 | <0.001 | 2.28 | 2.09 | 2.78 | 3.18 |
| 1306 | Annexin A2 | ANXA2 | 1.57 | <0.001 | <0.001 | 1.53 | 1.67 | 1.30 | 1.89 |
| 1417 | Annexin A4 | ANXA4 | 1.70 | <0.001 | <0.001 | 1.39 | 1.40 | 1.86 | 2.46 |
| 1596 | Apolipoprotein A1 | APOA1 | 1.99 | <0.001 | <0.001 | 1.03 | 2.09 | 2.49 | 3.24 |
| 1618 |  |  | -1.69 | <0.001 | <0.001 | -3.03 | -1.56 | -1.40 | -1.40 |
| 1631 |  |  | -1.51 | 0.001 | 0.005 | -2.22 | -1.26 | -1.39 | -1.35 |
| 1548 | Rho GDP-dissociation inhibitor 1 | ARHGDIA | -1.64 | <0.001 | <0.001 | -2.28 | -1.81 | -1.36 | -1.19 |
| 984 | ATP synthetase subunit beta | ATP5B | -1.51 | <0.001 | <0.001 | -1.61 | -1.28 | -1.58 | -1.78 |
| 873 | CCT5 chaperonin containing TCP1 | CCT5 | -1.39 | 0.010 | 0.043 | -1.47 | -1.20 | -1.23 | -1.69 |
| 1819 | Cofilin 1, non muscle | CFL1 | -1.30 | 0.012 | 0.046 | 1.04 | -1.18 | -1.85 | -1.31 |
| 1750 | CLEC3B C-type lectin domain family 3, member B | CLEC3B | 1.29 | 0.010 | 0.047 | -1.02 | 1.38 | 1.64 | 1.29 |
| 1466 | Chloride intracellular channel protein | CLIC1 | -1.39 | 0.002 | 0.012 | -1.09 | -1.75 | -1.35 | -1.47 |
| 1277 | Crk-like protein | CRKL | 1.70 | <0.001 | <0.001 | 1.82 | 1.92 | 1.30 | 2.32 |
| 1474 | Cathepsin D | CTSD | 1.32 | 0.010 | 0.046 | 1.99 | 1.18 | 1.00 | 1.34 |
| 926 | Desmin | DES | -2.0 | <0.001 | <0.001 | -1.69 | -1.64 | -2.32 | -2.63 |
| 931 |  |  | -2.94 | <0.001 | <0.001 | -2.05 | -2.32 | -3.85 | -4.54 |
| 938 |  |  | -2.13 | <0.001 | <0.001 | -1.72 | -1.67 | -2.39 | -2.86 |
| 1985 | Fatty acid binding protein, heart | FABP3 | 1.50 | 0.004 | 0.004 | 1.22 | 1.31 | 1.73 | 1.87 |
| 1999 |  |  | 1.49 | <0.001 | <0.001 | 1.21 | 1.23 | 1.90 | 1.79 |
| 2005 | Fatty acid binding protein, adipocyte | FABP4 | 2.24 | <0.001 | <0.001 | 1.89 | 1.65 | 2.98 | 2.84 |
| 2010 |  |  | 2.57 | <0.001 | <0.001 | 2.41 | 1.85 | 2.81 | 3.55 |
| 930 | Fascin | FSCN1 | -1.31 | 0.003 | 0.012 | -1.25 | -1.27 | -1.49 | -1.41 |
| 1218 | Galactokinase | GALK1 | 1.58 | <0.001 | <0.001 | 1.27 | 1.65 | 1.90 | 1.65 |
| 1256 | Aldose 1-epimerase | GALM | 1.17 | 0.029 | 0.073 | 1.17 | 1.31 | 1.20 | 1.03 |
| 612 | Gelsolin | GSN | 1.55 | <0.001 | <0.001 | 1.31 | 1.28 | 1.66 | 2.46 |
| 778 | 70 kDa heat shock protein 1B | HSPA1B | 1.32 | 0.002 | 0.010 | 1.14 | 1.04 | 1.83 | 1.50 |
| 754 | 71 kDa Heat shock cognate protein | HSPA8 | -1.44 | <0.001 | 0.002 | -1.72 | -1.28 | -1.47 | -1.32 |
| 853 | 60 kDa heat shock protein, mitochondrial | HSPD1 | 1.29 | 0.005 | 0.023 | 1.04 | 1.71 | 1.01 | 1.61 |
| 2022 | Lectin, galactoside binding | LGALS1 | -1.41 | 0.015 | 0.052 | -1.39 | -1.61 | -1.35 | -1.31 |
| 1346 | Lactate dehydrogenase B | LDHB | 1.44 | <0.001 | <0.001 | 1.17 | 1.33 | 1.43 | 1.92 |
| 1372 |  |  | 1.41 | <0.001 | <0.001 | 1.09 | 1.33 | 1.39 | 1.90 |
| 1370 | Malate dehydrogenase cytoplasmic | MDH1 | 1.58 | <0.001 | <0.001 | 1.65 | 1.61 | 1.72 | 1.41 |
| 1386 |  |  | 1.16 | 0.029 | 0.087 | 1.10 | 1.15 | 1.67 | 1.20 |
| 1796 | Myosin regulatory light chain 12A | MYL12A | 1.23 | 0.050 | 0.143 | 1.58 | 1.37 | -1.61 | 1.77 |
| 1609 | Protein L isoaspartate O-methyl transferase | PCMT1 | 1.17 | 0.006 | 0.031 | 1.48 | 1.00 | 1.16 | 1.08 |
| 1342 | Pyruvate dehydrogenase E1 subunit beta | PDHB | 1.21 | 0.014 | 0.051 | 1.17 | 1.04 | 1.29 | 1.33 |
| 988 | Protein disulfide isomerase A6 | PDIA6 | -1.37 | <0.001 | <0.001 | -1.44 | -1.06 | -1.61 | -1.37 |
| 1501 | Prohibitin | PHB | 1.35 | 0.004 | 0.018 | 1.06 | 1.86 | 1.27 | 1.40 |
| 1853 | Prefoldin | PFDN2 | -1.40 | <0.001 | 0.001 | -1.39 | -1.22 | -1.58 | -1.58 |
| 1665 | Peroxiredoxin-1 | PRDX1 | 1.17 | 0.010 | 0.050 | 1.18 | 1.42 | 1.09 | 1.04 |
| 1608 | Peroxiredoxin-1 | PRDX6 | 1.38 | 0.006 | 0.026 | 1.04 | 1.41 | 1.44 | 1.72 |
| 1223 | Prostaglandin reductase 2 | PTGR2 | 1.74 | 0.003 | <0.001 | 1.50 | 1.92 | 1.92 | 1.64 |
| 1191 | Transcriptional activator protein Pur-alpha | PURA | 1.50 | <0.001 | <0.001 | 1.29 | 1.44 | 1.74 | 1.68 |
| 1158 | Septin-2 | SEPT2 | 1.48 | <0.001 | <0.001 | 1.24 | 1.84 | 1.43 | 1.53 |
| 858 | Alpha-1-antitrypsin | SERPINA1 | -1.39 | <0.001 | <0.001 | -1.78 | -1.29 | -1.49 | -1.06 |
| 870 |  |  | -1.39 | <0.001 | <0.001 | -1.82 | -1.21 | -1.59 | -1.11 |
| 884 |  |  | -1.98 | 0.010 | 0.040 | -2.63 | -1.09 | -1.17 | -1.23 |
| 896 |  |  | -1.43 | 0.008 | 0.032 | -1.81 | -1.51 | -1.42 | -1.02 |
| 979 | HSP70-interacting protein | ST13 | -1.25 | 0.016 | 0.097 | -1.30 | -1.20 | -1.13 | -1.30 |
| 1858 | Stathmin | STMN1 | -1.92 | <0.001 | <0.001 | -2.04 | -1.49 | -1.77 | -2.59 |
| 1408 | Tropomyosin alpha-3 chain | TPM3 | 2.88 | <0.001 | <0.001 | 5.16 | 1.32 | 1.98 | 5.81 |
| 950 | Tubulin alpha 1B chain | TUBA1B | -2.29 | <0.001 | <0.001 | -2.27 | -1.85 | -2.27 | -3.16 |
| 963 | Tubulin beta chain | TUBB | -2.29 | <0.001 | <0.001 | -2.63 | -1.74 | -2.22 | -2.97 |
| 970 |  |  | -2.29 | <0.001 | <0.001 | -2.48 | -1.74 | -2.12 | -3.06 |
| 436 | Vinculin | VCL | -1.18 | 0.005 | 0.023 | 1.07 | -1.16 | -1.05 | -1.67 |
| 952 | Vimentin (fragments) | VIM | -1.78 | <0.001 | <0.001 | -2.04 | -1.49 | -1.76 | -1.82 |
| 1018 |  |  | -1.19 | 0.020 | 0.067 | -1.35 | -1.12 | -1.20 | -1.12 |
| 1047 |  |  | 1.62 | <0.001 | <0.001 | 1.42 | 1.51 | 1.47 | 2.34 |
| 1048 |  |  | 1.62 | <0.001 | <0.001 | 1.28 | 1.48 | 1.47 | 2.57 |
| 1077 |  |  | 1.38 | 0.002 | 0.009 | 1.33 | 1.32 | 1.08 | 1.95 |
| 1080 |  |  | 1.38 | <0.001 | 0.003 | 1.33 | 1.45 | 1.02 | 2.01 |
| 1043 |  |  | 1.41 | <0.001 | 0.002 | 1.15 | 1.42 | 1.32 | 1.93 |

^1^Mean ratio of values at d110 d values at d90 of gestation and ratios within each fetus genotype are shown. Ratios are inversed and are preceded by a minus sign for values less than 1

^2^P value for the effect of developmental age on abundance of the identified protein spots

^3^P value with Benjamini Hochberg (BH) correction for multiple tests
