# Supplementary material for: Proteomic analysis of adipose tissue during the last weeks of gestation in pure and crossbred Large White or Meishan fetuses gestated by sows of either breed
Source: J Anim Sci Biotechnol. 2018 Apr 3;9:28. doi: 10.1186/s40104-018-0244-2 (PMC5881184; doi:10.1186/s40104-018-0244-2)
Supplement: Supplementary file 6 — Proteins showing a differential abundance in adipose tissue according to fetus genotype. (DOCX 96 kb) [file 40104_2018_244_MOESM6_ESM.docx]

Additional file 6. Proteins showing a differential abundance in adipose tissue according to fetus genotype

| Spot | Protein name | Derived HUGO | P value^1^  Genotype | | Spot abundance |
| --- | --- | --- | --- | --- | --- |
| 1646 | Abhydrolase domain-containing protein 14B | ABHD14B | 0.001 | |  |
| 801 | Serum albumin | ALB | 0.004 | |  |
| 807 |  |  | 0.010 | |  |
| 809 |  |  | 0.030 | |  |
| 827 |  |  | 0.010 | |  |
| 855 |  |  | 0.005 | |  |
| 859 |  |  | 0.007 | |  |
| 866 |  |  | 0.010 | |  |
| 867 |  |  | 0.010 | |  |
| 1193 | Fructose biphosphate aldolase | ALDOC | 0.030 | |  |
| 1306 | Annexin 2 | ANXA2 | 0.010 | |  |
| 1607 | Apolipoprotein A1 | APOA1 | 0.008 | |  |
| 1618 |  |  | <0.001 | |  |
| 1631 |  |  | 0.010 | |  |
| 1548 | Rho GDP-dissociation inhibitor 1 | ARHGDIA | <0.001 | |  |
| 2233 | F-actin capping protein subunit beta | CAPZB | <0.001 | |  |
| 1466 | Chloride intracellular channel protein | CLIC1 | 0.045 | |  |
| 2037 | Cystatin B | CSTB | 0.010 | |  |
| 1985 | Fatty acid binding protein, heart | FABP3 | 0.047 | |  |
| 1999 |  |  | 0.020 | |  |
| 2010 | Fatty acid binding protein, adipocyte | FABP4 | <0.001 | |  |
| 612 | Gelsolin | GSN | 0.050 | |  |
| 778 | 70 kDa heat shock protein 1B | HSPA1B | | 0.010 |  |
| 1946 | Myosin light polypeptide 6, non-muscle | MYL6 | | 0.020 |  |
| 1796 | Myosin regulatory light chain 12A | MYL12A | | 0.010 |  |
| 1930 | Nucleoside diphosphate kinase A1 | NME1 | | 0.024 |  |
| 903 | Protein disulfide-isomerase A3 | PDIA3 | | 0.030 |  |
| 988 | Protein disulfide-isomerase A6 | PDIA6 | | 0.020 |  |
| 1853 | Prefoldin | PFDN2 | | 0.040 |  |
| 1608 | Periredoxin-6 | PRDX6 | | 0.025 |  |
| 356 | Gamma-tubulin complex component 2 | TUBGCP2 | | 0.030 |  |
| 368 | Spectrin alpha chain | SPTAN1 | | 0.001 |  |
| 668 | Transferrin | TF | | 0.030 |  |
| 670 |  |  |  | <0.001 |  |
| 675 |  |  |  | 0.05 |  |
| 771 |  |  |  | 0.002 |  |
| 1077 | Vimentin | VIM | | 0.050 |  |
| 1080 |  |  |  | 0.040 |  |

^1^P value for the effect of fetus genotype on abundance of the identified protein spots

^2^Relative values observed in Meishan (MeiS), Large White (LW) and crossbred (F1_MeiS in MeiS sows; F1_LW in LW sows) fetuses across gestation. Spot volume in each image obtained for a given fetus was expressed as a spot ratio of the internal standard which was a mix of all the analyzed samples and loaded onto all analyzed gels to allow accurate inter-gel protein spot comparisons Ratios were minus Log10 transformed in order to aid for graphical representation.
