# Supplementary material for: Proteomic analysis of adipose tissue during the last weeks of gestation in pure and crossbred Large White or Meishan fetuses gestated by sows of either breed
Source: J Anim Sci Biotechnol. 2018 Apr 3;9:28. doi: 10.1186/s40104-018-0244-2 (PMC5881184; doi:10.1186/s40104-018-0244-2)
Supplement: Supplementary file 7 — Proteins in adipose tissue affected by age in a different manner according to fetus genotype. (DOCX 32 kb) [file 40104_2018_244_MOESM7_ESM.docx]

Additional file 7. Proteins in adipose tissue affected by age in a different manner according to fetus genotype

| Spot | Protein name | Derived HUGO | P value^1^  AxG | LW^2^  110/90 | F1_LW  110/90 | MeiS 110/90 | F1_MeiS 110/90 |
| --- | --- | --- | --- | --- | --- | --- | --- |
| 798 | Albumin | ALB | 0.01 | -2.32 | -1.69 | 2.14 | 2.75 |
| 801 |  |  | 0.01 | -2.86 | -1.61 | 1.07 | 1.35 |
| 807 |  |  | 0.01 | -3.12 | -1.69 | 1.90 | 1.54 |
| 809 |  |  | 0.02 | -2.56 | -1.64 | 1.66 | 1.48 |
| 827 |  |  | 0.008 | -2.63 | -1.59 | 1.77 | 1.58 |
| 855 |  |  | 0.02 | -2.85 | -1.67 | 1.38 | 1.31 |
| 859 |  |  | 0.02 | -2.78 | -1.75 | 1.44 | 1.31 |
| 866 |  |  | 0.01 | -2.15 | -1.45 | 1.65 | 1.32 |
| 867 |  |  | 0.02 | -2.70 | -1.81 | 1.58 | 1.44 |
| 848 |  |  | 0.03 | -1.45 | -1.23 | -1.20 | 1.41 |
| 863 | Alpha-2-HS-glycoprotein (Fragment) | AHSG | 0.01 | -2.12 | -1.82 | 1 | 1.09 |
| 871 |  |  | 0.01 | -1.49 | -1.67 | 1.05 | 1.15 |
| 880 |  |  | 0.02 | -1.35 | -1.58 | 1 | 1.38 |
| 887 |  |  | 0.01 | -1.18 | -1.56 | -1.02 | 1.48 |
| 1065 |  |  | 0.02 | -2.04 | -2.38 | -1.05 | 1.66 |
| 1596 | Apolipoprotein A-I | APOA1 | 0.008 | -1.03 | 2.09 | 2.24 | 3.23 |
| 1607 |  |  | 0.01 | -1.07 | 1.15 | -1.35 | 1.69 |
| 1618 |  |  | 0.04 | -3.33 | -1.44 | -1.38 | -1.38 |
| 915 | Calreticulin | CALR | 0.04 | 1.66 | -1.45 | 1.20 | -1.42 |
| 573 | Endoplasmin | HSP90B1 | 0.03 | -1.33 | 1.44 | -1.33 | 1.12 |
| 612 | Gelsolin | GSN | 0.04 | 1.29 | 1.29 | 1.70 | 1.51 |
| 1106 | Isocitrate dehydrogenase | IDH1 | 0.01 | -1.57 | -1.09 | 1.64 | -1.04 |
| 1796 | Myosin regulatory light chain 12A | MYL12A | 0.009 | 1.58 | 1.35 | -1.59 | 1.69 |
| 1733 | Phosphatidylethanolamine-binding protein | PEBP1 | 0.04 | 1.54 | 1.54 | 1.12 | -2.63 |
| 666 | Serotransferrin | TF | 0.03 | -1.78 | 1.74 | -1.23 | 2.13 |
| 667 |  |  | 0.05 | -1.56 | 1.51 | -1.15 | 2.04 |
| 667 |  |  | 0.05 | -1.56 | 1.51 | -1.15 | 2.04 |
| 668 |  |  | 0.05 | -1.17 | 1.48 | -1.51 | 1.55 |
| 670 |  |  | 0.07 | -1.41 | 1.38 | 1 | 1.61 |
| 675 |  |  | 0.01 | -1.56 | 1.66 | 1.07 | 1.74 |
| 771 |  |  | 0.04 | -1.49 | 1.15 | -1.75 | 1.69 |
| 848 | Serpin family A member 1 | SERPINA1 | 0.03 | -1.44 | -1.23 | -1.20 | 1.41 |
| 1408 | Tropomyosin alpha-3 chain | TPM3 | 0.04 | 5.12 | 1.29 | 1.99 | 5.62 |
| 436 | Vinculin | VCL | 0.002 | 1.07 | -1.17 | -1.03 | -1.67 |

^1^P value for the interaction effect between developmental age and fetus genotype (AxG) on abundance of the identified protein spots

^2^Relative values observed in Meishan (MeiS), Large White (LW) and crossbred (F1_MeiS in MeiS sows; F1_LW in LW sows) fetuses between d90 and d110 of gestation
